# Sociodemographic Correlates of Food Insecurity in Students Attending a Private University: A Cross-Sectional, Descriptive Study

**DOI:** 10.7759/cureus.28987

**Published:** 2022-09-09

**Authors:** Danielle Lankford, Jessica Bernstein, Angelica Green, Nadine Mikati, Stephanie N Petrosky, Robin J Jacobs

**Affiliations:** 1 Nutrition, Nova Southeastern University Dr. Kiran C. Patel College of Osteopathic Medicine, Fort Lauderdale, USA; 2 Medical and Behavioral Research; Health Informatics; Medical Education, Nova Southeastern University, Fort Lauderdale, USA

**Keywords:** campus wellness, student financial aid, private university, college students, food insecurity

## Abstract

Background: Food insecurity is thought to be a prevalent yet misunderstood problem among college/university students. There is limited evidence regarding the prevalence of food insecurity in this population, and even less among private institutions of higher education. Food insecurity in college/university students can have harmful effects on students’ academic performance and health. The aim of this study was thus to examine food security among college/university students and assess variables which may be associated with it.

Methods: Cross-sectional data were collected during October-November 2021 from undergraduate, graduate, and professional students at a large private university in Florida, United States using an anonymous, self-administered online questionnaire that included the Validate U.S. Department of Agriculture Household Food Security Six-item index and select sociodemographic variables. Point-biserial correlation coefficient analysis was conducted to identify correlations between the scores on the U.S. Household Food Security Six-item Index and sociodemographic variables. Data were analyzed using SPSS v.27 (IBM Corp., Armonk, NY).

Results: Among the 1,316 students who completed the questionnaire, 34% (n=447) reported being “food insecure” (scores between 2 and 6), with higher scores indicating more low food security. In addition, there was a weak but statistically significant correlation between identifying as a first-generation college/university student and greater food insecurity. Conversely, current employment and receiving financial aid from family were both weakly, yet statistically significantly correlated with less food insecurity.

Conclusion: Results from this study may help raise awareness regarding university students at risk for low food security and understand certain socioeconomic variables associated with high levels of food insecurity. More research may be needed to help direct focused intervention efforts such as food assistance programs and on-campus food pantries to ameliorate food insecurity in college/university students.

## Introduction

Food insecurity has been defined by the United States Department of Agriculture (USDA) as “a lack of consistent access to enough food for an active, healthy life [1]. While hunger and food insecurity are closely related, they are discrete concepts. Hunger is physical sensation of distress, while food insecurity denotes a lack of available financial resources for food at the household level.

Food insecurity, specifically among college and university students, has received little attention in the scientific literature. About 13.7 million households (10.5%) have experienced food insecurity, and since the coronavirus disease 2019 (COVID-19) pandemic, this number has more than doubled; some of these household also include college/university students at the undergraduate and graduate levels [2]. Students are especially susceptible to food insecurity as they transition into their new-found autonomy during this period [3]. Their budgets for rent, tuition, and utilities may leave a deficit in food budgets, which can lead to risk of food insecurity [3]. 

Among these students, a large demographic of first-generation college students who seek degrees exist [4]. First-generation college students report a slightly higher average of food insecurity than non-first-generation college students [5]. First-generation students are already less likely to persist in college than their non-first-generation peers [6] without this additional burden of having access to nutritious food.

Another trend that leads to food insecurity in college students is being employed while in school. In a competitive labor market where the value of minimum wage has decreased coupled with rising college prices, a majority of students must work nearly full-time [7]. Work schedules and class schedules often clash as well. While working, students have less flexibility when it comes to shift changes and may have to skip classes leading to earning less money and lower grades [7].

Unfortunately, very few students are eligible to receive food assistance from the Supplemental Nutrition Assistance Program (SNAP). Few students are eligible due to the program’s rules which states that students can only be included on parents’ SNAP grants if eat at least half their meals at home [7]. Students who are food insecure are less likely to earn high grades on coursework, while other consequences from food insecurity include a decrease in completion, persistence, and credit attainment rates [3]. Moreover, food insecurity has been known to be associated with depression, diabetes, obesity, hypertension, poor sleep, and lower self-rated health [8].

Private universities are less represented than community colleges and public universities with regard to awareness about student food insecurity [9]. There appears to be an assumption that students attending private educational institutions are more financially secure than those attending public colleges/universities as private schools tend to have higher tuition rates. This assumption contributes to the stigma regarding poverty that some students with food insecurity experience [10]. Because the cost of tuition and fees are typically higher at private colleges [11], students have less funds to allocate to food. Even at the nation’s wealthiest institutions, lower-income students often struggle to purchase food, particularly during school closures (e.g., holidays, spring, and winter breaks), when dorms and dining halls often close [12]. According to US federal guidelines, most college students are not eligible for the SNAP [13]. While food pantries have become one of the fastest growing forms of emergency relief on college campuses and are designed to decrease food insecurity among students, some of these services may not be known to faculty and/or students.

In a study conducted at a mid-sized private, catholic university [14], student demographics, food security, financial priority, and nutrition literacy were assessed in 560 students. Their findings revealed that 63.3% of students were food secure, 25.4% classified as low food secure, and 11.3% as very low food secure. A large, public university in Florida, United States found that 24% of those students surveyed had skipped a meal before because they could not afford food and about 14% have needed assistance in acquiring food [15]. A study conducted with eight private and public US universities, found that 19% were food-insecure while an additional 25.3% were at risk of food insecurity [11].

There is limited published evidence regarding the prevalence of and sociodemographic variables related to food insecurity among private US academic institution students. This research attempted to address this knowledge gap by investigating the level of food security and its relationship to sociodemographic variables in students attending a large, private US university. It was hypothesized that 1) there would be a prevalence of food insecurity and 2) there would be statistically significant correlations between food insecurity, select sociodemographic variables, and financial assistance in students enrolled in a large private US university.

## Materials and methods

The instrument

The research team developed a 22-item questionnaire that contained the validated measure US Household Food Security Module: Six-Item Short Form to investigate the prevalence of food insecurity in university students and 16 sociodemographic items. Food insecurity was assessed for the previous 12 months via the U.S. Department of Agriculture (USDA) Household Food Security Six-Item Index. This shortened tool has been validated with high specificity, sensitivity, and minimal bias [16]. The first two items, “The food I/we bought just didn’t last, and I/we didn’t have money to get more” and “I/We couldn’t afford to eat balanced meals” were scored using a Likert-type response set (often true, sometimes true, never true). The third item, “In the last 12 months, since last August, did you ever cut your meals or skip meals because there wasn’t enough money for food?” was scored using the response set yes, no, I don’t know. The fourth item, “How often did this happen? was a follow up question to the previous item (if the respondents answered “yes”) using the response set almost every month, some months but not every month, only one or two months. Items five and six, “In the last 12 months, did you ever eat less than you felt you should because there wasn’t enough money for food?,” and “In the last 12 months, were you ever hungry but didn’t eat because there wasn’t enough money for food?” were scored using the response set yes, no, I don’t know.

Based upon USDA household food security six-item form scoring instructions, food security status was assigned as follows: raw score 0-1 = high or marginal food security (food secure); raw score 2-4 = low food security (food insecure); and raw score 5-6 = very low food security (food insecure) when dichotomized.

In addition, questions related to personal characteristics (i.e., age, sex/gender, ethnicity, race); family education (i.e., highest education level of mother and father); student education (i.e., current academic level, full or part time status, if they were a first-generation college student, if they reside on campus, employment status (none, full, or part-time); and financial assistance (i.e., if they were a recipient of financial aid, have a university dining plan, receive financial aid from family, receive assistance from any food assistance programs) were included.

Recruitment 

A cross-sectional design was used to collect data from students enrolled at a large private not-for profit university in Florida, United States using an online, voluntary quantitative questionnaire. The questionnaire was distributed via student listservs to all enrolled students during October 2021. A paper flyer was also posted in strategic locations around campus distribution with information regarding the survey and a Quick Response (QR) code with a link to direct access to the survey.

The study was approved by the researchers’ university internal review board for research with human subjects. Participants were informed about the study through a cover letter that accompanied the questionnaire which contained a link to start the survey (indicating consent to participate). Participants gave consent to the survey through two opt-in questions prior to beginning the survey. Reminder emails were sent to potential participants at certain intervals to promote engagement.

Data analysis

The IBM SPSS Statistics for Windows v. 27 (IBM Corp., Armonk, NY) was used to analyze the data from this study [17]. Sample characteristics were summarized as frequency and percentage for discrete variables. The responses to the six items on the food insecurity scale were categorized as high food security (score 0), marginal food security (score 1), low food security (score 2-4), and very low food security (score 5 to 6), based upon USDA guidelines. The items were scored and summed to generate each respondent’s raw score. The raw scores (ranging from 0 to 6) were used to dichotomize the sample into “food insecure” and “food secure” households. After evaluating scores for food insecurity/security, socioeconomic characteristics expected to be correlated with food insecurity were examined. Point-biserial correlation coefficient analysis was conducted to identify correlations between the scores on the US Household Food Security Six-Item Index and sociodemographic variables.

## Results

The electronic survey was sent via email to 20,941 students enrolled at Nova Southeastern University) in Florida, United States. Of those, 1,334 questionnaires were returned (5.4% response rate). From those, 19 cases were deleted due to having less than 66% data submitted, resulting in a total of 1,316 completed questionnaires (98% completion rate).

Sample characteristics

The mean age of the participants was 25.9 years (range 18-73; standard deviation, SD=9.33). Table 1 reports the characteristics of the sample.

**Table 1:** Characteristics of the sample (n=1,316).

		n	%
Gender			
	Female	1,058	80.9
	Male	232	17.7
	Transgender	2	0.2
	Non-binary	11	0.8
	Prefer not to answer	12	0.9
Race			
	White	871	66.2
	Black/African American	179	13.6
	Asian	131	10
	Native American/Alaska Native	7	0.5
	Native Hawaiian/Pacific Islander	7	0.5
	Other/missing	121	9.2
	Hispanic ethnicity	429	32.6
First generation college student		421	32
Current academic level			
	First-year undergraduate student	220	16.7
	Second-year student	122	9.3
	Third-year undergraduate student	137	10.4
	Fourth-year undergraduate student	92	7
	Graduate student	560	42.6
	Professional student (e.g., M.D., D.O.)	184	14
Full-time student		1,185	90
Part-time student		130	10
Reside on campus		253	19.2
Currently employed		612	46.5
	Full-time	215	16.3
	Part-time	394	29.9
Financial aid recipient		917	69.7
Has a university dining plan		243	18.5
Receives financial assistance from family		668	52
Receives aid from food assistance programs		90	6.8

Food insecurity 

Regarding scores on the USDA Household Food Security Six-Item Index, 66% (n=869) of participants reported being food secure (scores 0-1, little or no food insecurity). “Food insecurity” was found in approximately one-third (n=447; 34%), with a mean score of 4.23 (SD=0.572) for the participants who scored between 2 and 6 (food insecure), whereby 6 is the maximum for “low food security.” Among the 447 participants who scored as being “food insecure,” 14.3% (n=64) scored a raw score of 5 and 33.8% (n=151) scored a raw score of 6 (highest level of food insecurity). Table 2 reports the frequencies of the food insecurity items for this group. 

**Table 2:** Frequencies of food insecurity items in the food insecure group (n=447).

Item	Yes (n, %)				
The food I/we bought just didn’t last, and I/we didn’t have money to get more.	420 (31.9)	---	---	---	---
I/We couldn’t afford to eat balanced meals.	439 (33.4)	---	---	---	---
In the last 12 months, did you ever eat less than you felt you should because there wasn’t enough money for food?	332 (25.8)	---	---	---	---
In the last 12 months, were you ever hungry but didn’t eat because there wasn’t enough money for food?	263 (25.2)	---	---	---	---
In the last 12 months, since last August, did you ever cut your meals or skip meals because there wasn’t enough money for food?	340 (25.8)	---	---	---	---
		Almost every month (n, %)	Some months (n, valid %)	Only 1 or 2 months (n, valid %)	Don’t know (n, valid %)
----- How often did this happen?	340 (25.8)	125 (36.8)	129 (39.9)	66 (19.4)	20 (4.9)

To further illustrate prevalence of participants who reported very high food insecurity, the 446 participants who reported some level of food insecurity (scores 2-6) were dichotomized into two groups; 51.9% (n=232) fell into the lower end of food insecurity (scores 2-4), while 41.1% (n=215) fell into the group with the highest high food insecurity (scores 5-6).

Food insecurity by academic level

Table 3 reports frequencies of students who scored as having some level of food insecurity using the USDA Food Insecurity Index by academic level/program.

**Table 3:** Students with food insecurity by academic level (n=477).

		First year	Second year	Third year	Fourth year	Graduate student	Professional student	Total
High food insecurity	Count (n)	48	29	30	19	80	26	232
	Percent within high food insecurity levels	20.70%	12.50%	12.90%	8.20%	34.50%	11.20%	100.00%
	Percent within academic level	57.80%	58.00%	44.10%	48.70%	51.00%	52.00%	51.90%
	Percent of total	10.70%	6.50%	6.70%	4.30%	17.90%	5.80%	51.90%
Very high food insecurity	Count (n)	35	21	38	20	77	24	215
	Percent within very high food insecurity levels	16.30%	9.80%	17.70%	9.30%	35.80%	11.20%	100.00%
	Percent within academic level	42.20%	42.00%	55.90%	51.30%	49.00%	48.00%	48.10%
	Percent of total	7.80%	4.70%	8.50%	4.50%	17.20%	5.40%	48.10%

Variables correlated with food insecurity

Weak but statistically significant correlations were found between food insecurity and the following socioeconomic variables (see Table 4). Being a first-generation college student was correlated with greater food insecurity (r = 0.121, p < 0.01); being currently employed (r = 0.078, p < 0.05) and receiving financial aid from family was correlated with less food insecurity (r = 0.176, p < 0.01). No statistically significant correlations were found between any of the other sociodemographic variables and food insecurity scores. 

**Table 4:** Correlations between food insecurity and sociodemographic variables. *p < 0.01

Food insecurity	
First generation student	-0.121^*^
Employment	-0.078^*^
Financial assistance from family	0.176^*^

## Discussion

The results of the survey support the hypothesis that food insecurity is prevalent among students who attended a large-size private university in Florida. When comparing the level of food insecurity in university students and non-university students in the United States, food insecurity is proportionately higher at the college/university level, identifying a concern among this population. In this sample, a significant number of students reporting food insecurity were found who may be at risk for adverse consequences in academic performance and health outcomes. 

The findings also indicate that food insecurity may be a tangible threat in private universities and not isolated to schools in low-income areas or under a public funding system. Further, of the 447 students who reported some level of food insecurity, nearly 34% scored at the highest level. Another unique attribute is the finding that the highest proportion of students in the food insecure group was graduate students at 34.5% followed by first-year student at 20.7%. While we anticipated the first-year students to be most likely, the distribution in all levels was unexpected.

Regarding sociodemographic correlations with food insecurity, it was not surprising that being a first-generation college student and being unemployed was associated with food insecurity in participants. Unsurprisingly, first-generation students face special risks overall, such as coming from families with lower incomes, tending to leave college without finishing because they cannot afford tuition, as compared to students from families where at least one parent had earned a bachelor’s degree.

Studies conducted at private and public universities have reported similar results regarding food insecurity [11, 14-17]. However, variations in the sampling methods might impact the usefulness of interpretation. For example, in one study, to be eligible to participate, students had to self-report consuming less than two cups of fruit and/or three cups of vegetables and have at least one risk factor for weight gain such as body mass index (BMI) equal to or more than 25 kg/m2 or have a parent who is overweight or obese [11]. It is possible that students who were already sensitive to the food security topic chose to respond to the survey out of personal interest. Thus, our data may be skewed in favor of this preference. 

Published research investigating food insecurity among college students is scant, and less has been investigated with private universities. Students who attend private universities are often assumed to have little or no problems with food security due the stereotype that private school students have more financial resources. Low-income students are able to attend top universities through financial aid and scholarships. In addition to tuition, food and other miscellaneous costs are potential burdens on students [9]. Students may be juggling newfound social engagement, peer interactions, academic expectations, and feelings of belonging [9]. Future exploration including academic performance or overall health in life should be considered with its impact on college students.

Implications for practice

Results from this study may be used to advocate for more university involvement in combating food insecurity on campus. Programs such as campus food pantries, food assistance during university breaks, and allocation of more financial aid to offset food costs could lighten the burden on students. Institutions could implement education programs focused on students wanting to increase their financial literacy to budget their financial aid or provide more information on government assistance programs including SNAP. Participation from the community through local partnerships, awareness campaigns, and sponsorships would provide additional resources to ease qualifications for SNAP benefits and/or bring awareness to issues students encounter while applying for government assistance programs such as SNAP. Few students are enrolled in SNAP as the program intentionally tries to keep college students from using it. However, even though there are some rules that do allow students to qualify for SNAP, the process of applying can be confusing and uncertain.

Limitations of the study

A cross-sectional, correlational survey design was used to collect data from a single, private university and generalizations cannot be made concerning changes over time. Moreover, due to the type of study, causal relationships between variables cannot be determined. As a single-site study, it is improbable that the data reflect a diverse sample of student participants. Moreover, self-report questionnaires may produce response bias, social desirability bias, and other inaccuracies. Additionally, there was a relatively low response rate (about 5%) compared to the rate for the university’s annual student survey (18%-20%). Moreover, the social and economic effects of COVID-19 (e.g., stress, isolation, job loss or cut in hours) may have influenced participant responses. However, due to the large sample size, the conclusions drawn from this survey may be of some use in directing more focused research efforts to reveal the prevalence of food insecurity in university students, including associated sociodemographic variables.

## Conclusions

This study investigated the prevalence of food insecurity among undergraduate, graduate, and professional students at a large private US university. Findings from this study may provide insights into not only the high prevalence of food insecurity that may exist in private universities, but some of the sociodemographic and economic factors influencing this phenomenon. More research is needed to identify other variables that may influence food insecurity among college students not identified in this study, such as financial priorities and nutrition literacy. If anything, this study highlights the existence of food insecurity in university students which may in turn influence policy and initiatives aimed to eradicate food insecurity among college campuses nationwide.
